# Inflammation in multiple sclerosis induces a specific reactive astrocyte state driving non‐cell‐autonomous neuronal damage

**DOI:** 10.1002/ctm2.837

**Published:** 2022-05-11

**Authors:** Clara Matute‐Blanch, Verónica Brito, Luciana Midaglia, Luisa M Villar, Gerardo Garcia‐Diaz Barriga, Alerie Guzman de la Fuente, Eva Borrás, Sara Fernández‐García, Laura Calvo‐Barreiro, Andrés Miguez, Lucienne Costa‐Frossard, Rucsanda Pinteac, Eduard Sabidó, Jordi Alberch, Denise C. Fitzgerald, Xavier Montalban, Manuel Comabella

**Affiliations:** ^1^ Servei de Neurologia‐Neuroimmunologia, Centre d'Esclerosi Múltiple de Catalunya (Cemcat). Institut de Recerca Vall d'Hebron (VHIR) Hospital Universitari Vall d'Hebron. Universitat Autònoma de Barcelona Barcelona Spain; ^2^ Departament de Biomedicina, Facultat de Medicina Institut de Neurociències Universitat de Barcelona Barcelona Spain; ^3^ Institut d'Investigacions Biomèdiques August Pi i Sunyer (IDIBAPS), Hospital Clínic Universitat de Barcelona Barcelona Spain; ^4^ Centro de Investigación Biomédica en Red sobre Enfermedades Neurodegenerativas (CIBERNED) Madrid Spain; ^5^ Departments of Neurology and Immunology Hospital Universitario Ramón y Cajal, Instituto Ramón y Cajal de Investigacion Sanitaria Madrid Spain; ^6^ Wellcome‐Wolfson Institute for Experimental Medicine Queen's University Belfast, Belfast, UK; ^7^ Proteomics Unit, Universitat Pompeu Fabra (UPF) Barcelona Spain; ^8^ Proteomics Unit, Centre de Regulació Genòmica (CRG) Barcelona Institute of Science and Technology (BIST) Barcelona Spain

Dear Editor

An in‐depth understanding of the neurodegenerative component of multiple sclerosis (MS) is crucial for the design of therapeutic approaches that may stop disease progression. Astrocytes have emerged as key contributors to the pathogenesis of MS.^1^ However, the mechanisms underlying the regulation of maladaptive astrocytic responses remain unknown. In this report, we show that a high inflammatory activity in MS patients at disease onset induces a specific reactive astrocyte state that triggers synaptopathy and contributes to neuronal damage in vitro and ex vivo suggesting potential mechanisms that may ultimately lead to neurodegeneration.

To investigate whether astrocytes are essential contributors to neuronal damage in MS, we cultured purified astrocytes with cerebrospinal fluid (CSF) samples from MS patients with high inflammatory activity at disease onset (MS‐High, Table S1). Then, we examined the effect of astrocytic secretomes on neurons (Figure 1A). Astrocytes became reactive upon high inflammatory CSF exposure (Figure 1B) and induced morphological alterations typically observed in neurodegenerative disorders, such as a less complex dendritic tree due to decreased arborisation (Figure 1C, D). Moreover, these abnormalities were accompanied with synaptic plasticity impairment (Figure 1E, F). Considering that a high lesion load at disease onset has been associated with an increased risk of neurological disability development,^2^ we assessed whether the non‐cell‐autonomous effect on neuronal plasticity could be influenced by the degree of inflammatory activity of MS patients (Figure 2A and Table S1). Interestingly, we observed a direct correlation between the degree of inflammatory exposure and the extent of both astrocyte‐mediated synaptopathy (Figure 2B, C) and dendrite arborisation impairment (Figure 2D, E).

**FIGURE 1:**
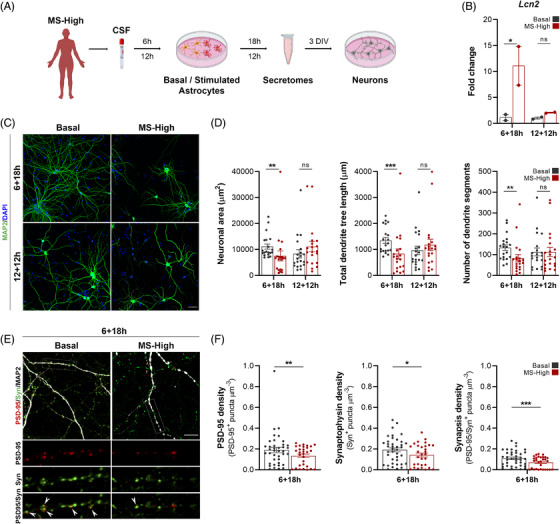
Astrocytes exposed to CSF from MS patients with high inflammatory activity induce synaptic plasticity impairment. (A) Flowchart depicting CSF collection from a cohort of MS patients with high inflammatory activity (MS‐High; *N *= 4), followed by exposure of astrocytes to the CSF in vitro, secretome collection and co‐culture with neurons. (B) qPCR for the astrocyte reactive marker lipocalin‐2 (*Lcn2*) of primary astrocytes exposed for 6 and 12 h to medium (Basal) or CSF. One‐way ANOVA analysis; *n *= 2 independent experiments. (C) Illustrative confocal images of primary cortical neurons treated with media (Basal) or MS‐High‐exposed astrocyte secretomes. Neurons were immunostained with MAP2 (green) and DAPI (blue). (D) Graphs represent individual data of neuronal area, total dendrite tree length and number of dendrite segments per neuron. Least Squares Means Estimates test, *n *= 2 replicates per condition, *n *= 2 independent experiments. (E) Illustrative confocal images of primary cortical neurons treated with Control or MS‐High‐exposed astrocyte secretomes. Neurons were immunostained with MAP2 (white), the pre‐synaptic marker Synaptophysin (green) and the post‐synaptic marker PSD‐95 (red). Arrows in high‐magnification insets point to co‐localization of Synaptophysin and PSD‐95 (synapses). (F) Graphs represent individual data of the density of PSD‐95, Synaptophysin and PSD‐95/Synaptophysin double‐positive puncta. Least Squares Means Estimates test; *n *= 40 neurons per condition, *n *= 2–3 dendrites per neuron, *n *= 2 independent experiments. Data are shown as mean (standard error of the mean, SEM). *p < .05, **p < .01, ***p < .001. Scale bars: 40 μm (B) and 10 μm (D). DIV: days in vitro

**FIGURE 2:**
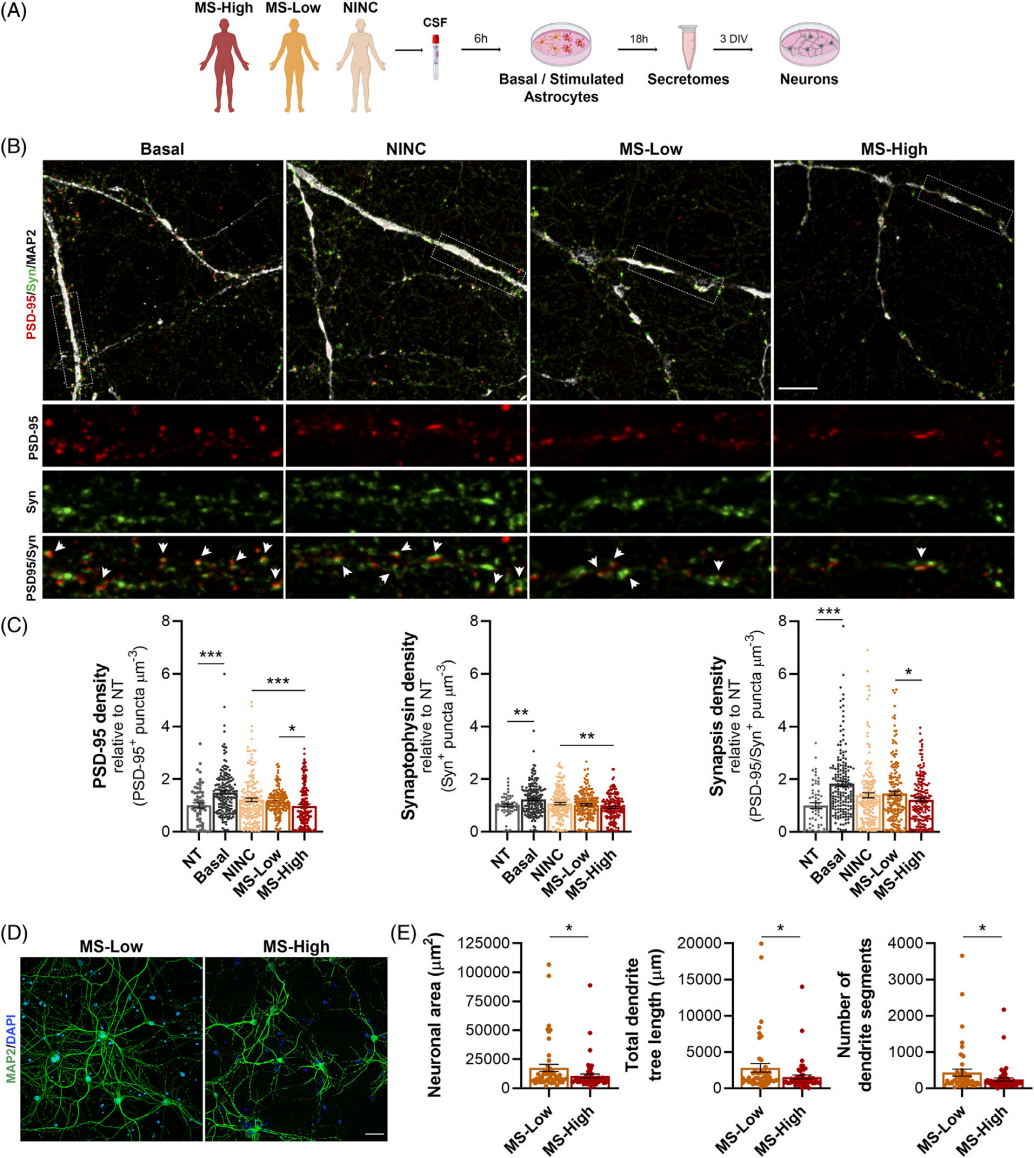
Astrocyte‐mediated synaptic plasticity impairment correlates with the degree of inflammatory activity in MS patients. (A) Flowchart depicting CSF collection from a cohort of MS patients with high inflammatory activity (MS‐High; *N* = 9), MS patients with low inflammatory activity (MS‐Low; *N* = 9) and non‐inflammatory neurological controls (NINC; *N* = 9) followed by exposure of astrocytes to the CSF in vitro, secretome collection and co‐culture with neurons. (B) Illustrative confocal images of primary cortical neurons treated with secretomes from astrocytes exposed to CSF. Neurons were immunostained with MAP2 (white), the pre‐synaptic marker Synaptophysin (green) and the post‐synaptic marker PSD‐95 (red). Arrows in high‐magnification insets point to co‐localisation of Synaptophysin and PSD‐95 (synapses). (C) Graphs represent individual relative numbers of PSD‐95, Synaptophysin and PSD‐95/Synaptophysin double‐positive puncta density normalised to untreated neurons (NT). Least Squares Means Estimates test, Tukey–Kramer multiple comparisons test; *n *= 3 independent secretome samples per group, *n *= 180 neurons per condition (*n *= 60 for non‐treated neurons), *n *= 2–3 dendrites per neuron, *n *= 2 replicates per condition, *n *= 3 independent experiments. (D) Illustrative confocal images of primary cortical neurons treated with MS‐High and MS‐Low‐exposed astrocyte secretomes. Neurons were immunostained with MAP2 (green) and DAPI (blue). (E) Graphs represent individual data of neuronal area, total dendrite tree length and the number of dendrite segments per neuron. Least Squares Means Estimates test; *n *= 3 independent secretome samples per group, *n *= 2 replicates per condition, *n *= 2 independent experiments. Data are shown as mean (SEM). *p < .05, **p < .01, ***p < .001. Scale bars: 10 μm (B) and 40 μm (D). DIV: days in vitro

We next characterised the secretomes from astrocytes exposed to high inflammatory MS microenvironment and found an altered pro‐inflammatory profile comprised of 23 upregulated factors (Figure 3A). Functional enrichment and interactome analysis revealed a set of pro‐inflammatory pathways enriched following the MS‐High CSF exposure (Figure S1). Moreover, nuclear factor NF‐kappa‐B p105 subunit (*Nfkb1*) and cellular tumour antigen p53 (*Trp53*) were identified as the transcription factors regulating the MS‐High‐associated astrocyte secretome (Figure 3B), both involved in NF‐ĸB signalling.

**FIGURE 3:**
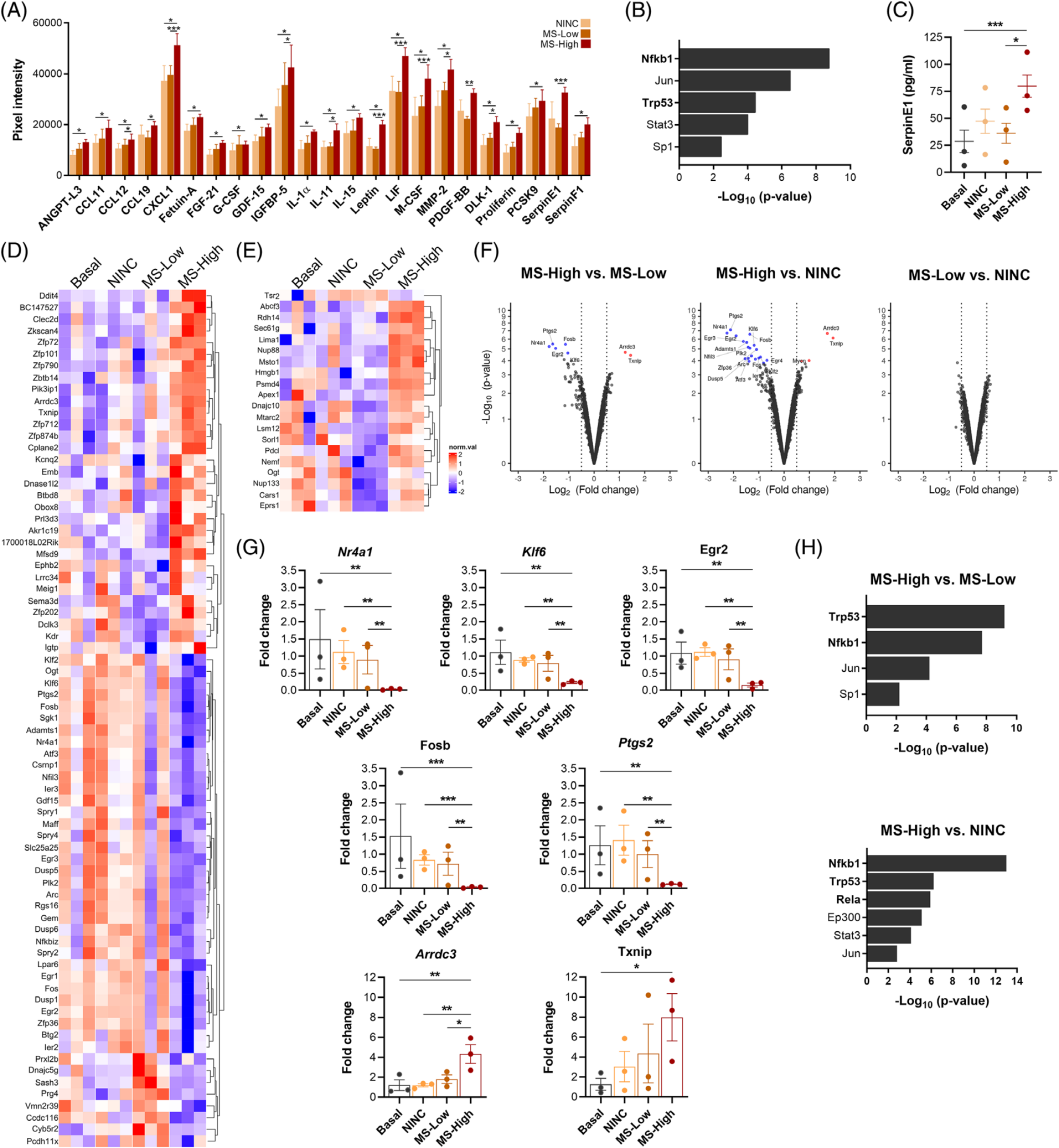
Astrocytes exposed to CSF from MS patients with high inflammatory activity have a specific reactive astrocyte state in vitro. (A) Proteome profiler array of secretomes from CSF‐exposed primary astrocytes showing 23 inflammation‐related molecules upregulated in the MS‐High condition. FDR analysis. *n *= 3 independent secretome samples per group, *n *= 3 independent experiments. (B) TRRUST enrichment analysis identifying *Nfkb1* as a candidate key transcription factor modulator of upregulated astrocyte‐secreted factors in the MS‐High condition (*FDR *= 1.9 × 10^–8^). (C) Dot plot showing SerpinE1 levels determined by ELISA in astrocyte‐derived secretomes. Least Squares Means Estimates test and Tukey–Kramer multiple comparisons test; *n *= 3 independent secretome samples per group. (D–F) Gene expression microarrays and liquid chromatography/mass spectrometry analysis of reactive astrocytes exposed to CSF from MS patients with high inflammatory activity (MS‐High), low inflammatory activity (MS‐Low) and non‐inflammatory neurological controls (NINC). (D, E) Heatmaps and (F) volcano plots showing normalised Log_2_ gene (D, F) and protein (E) expression satisfying *p* value < .01 and absolute *FC* > 0.5; *n *= 3 independent biological samples per group. FDR analysis; *n *= 3 independent biological samples per group. (G) mRNA expression levels measured by qPCR of the specific gene expression signature associated with astrocyte exposure to CSF from MS‐High patients. Individual values represent average *FC* = 2^–(average ∆∆Ct)^ in mRNAs of CSF‐exposed astrocytes relative to non‐stimulated astrocytes (Basal). Least Squares Means Estimates test and Dunnett–Hsu test for multiple comparisons; *n *= 3 independent biological samples per group. (H) TRRUST enrichment analysis identifying *Nfkb1*, *Trp53* and *Rela* as candidates key transcription factor modulator of MS‐High‐reactive astrocyte signature. Data are shown as mean (SEM). *p < .05, **p < .01, ***p < .001

SerpinE1, also known as plasminogen activator inhibitor 1 (PAI‐1), which has been shown to exacerbate axonal damage and demyelination in MS animal models^3^ and be regulated by NF‐ĸB in neuroinflammation,^4^ was significantly increased in the MS‐High secretomes (Figure 3A and Table S2). Considering its potential role as a mediator of neurodegeneration, we validated by ELISA SerpinE1 increased levels in secretomes from astrocytes exposed to MS‐High condition (Figure 3C).

By using omics technologies, we studied whether secretomes that alter neuronal plasticity are associated with a specific reactive astrocyte state in MS patients with high inflammatory activity. Astrocytes stimulated with MS‐High CSF exhibited a specific reactive gene (Figure 3D) and protein (Figure 3E) expression profile. We identified a MS‐High‐associated reactive gene signature comprised of 7 differentially expressed genes (Figure 3F) that were validated by qPCR (Figure 3G). This reactive gene expression fingerprint was mostly comprised of downregulated immediate early response genes (*Nr4a1*, *Klf6*, *Egr2* and *Fosb*). Interestingly, *Nr4a1* and *Klf6* have been reported to promote anti‐inflammatory responses by specifically repressing NF‐ĸB activity.^5^, ^6^ To further decipher the MS‐High‐specific reactive astrocyte state, we performed a functional enrichment analysis integrating all datasets obtained from CSF exposed astrocytes: secretomes and gene/protein expression. Overall, this revealed a prominent inflammatory signature in MS‐High astrocytes (Figure S2). Furthermore, *Nfkb1*, *Trp53* and transcription factor p65 (*Rela*), were identified as the transcription factors regulating the MS‐High astrocyte‐specific fingerprint (Figure 3H). The inhibition of NF‐кB activation in astrocytes ameliorated immune infiltrate, axonal damage and demyelination, by preventing the establishment of the astrocyte‐mediated pro‐inflammatory microenvironment that leads to disease progression in EAE.^7^, ^8^ Moreover, a common MS risk variant (rs7665090) has been found associated to increased NF‐кB signalling in astrocytes, driving increased lymphocyte infiltrate and lesion size.^9^ These findings provide evidence that a high inflammatory microenvironment in MS patients may mediate disease progression by enhancing NF‐кB signalling in astrocytes, which modifies their secretome content resulting in both immune‐mediated neurodegeneration and potential direct neurotoxic effects.

Next, we investigated whether this reactive astrocyte state is associated with a specific CSF proteome in MS patients with high inflammatory activity. LC/MS analysis showed that CSF from MS‐High patients have a specific proteome profile (Figure 4A, B). To elucidate the mechanisms underlying astrocyte reactivity, we performed an integrative omics data analysis at CSF, reactive astrocytes, and secretomes levels. Mitogen‐activated protein kinase (ERK)‐1/2 cascade, NF‐ĸB‐inducing kinase (NIK)/NF‐ĸB signalling, *Nfkb1* and *Trp53* were found as the most significantly enriched pathways and transcription factors by the exposure to a highly inflammatory MS microenvironment (Figure 4C and Table S3). These data reinforced the role of an enhanced NF‐ĸB signalling in the MS‐High reactive astrocytes.

**FIGURE 4:**
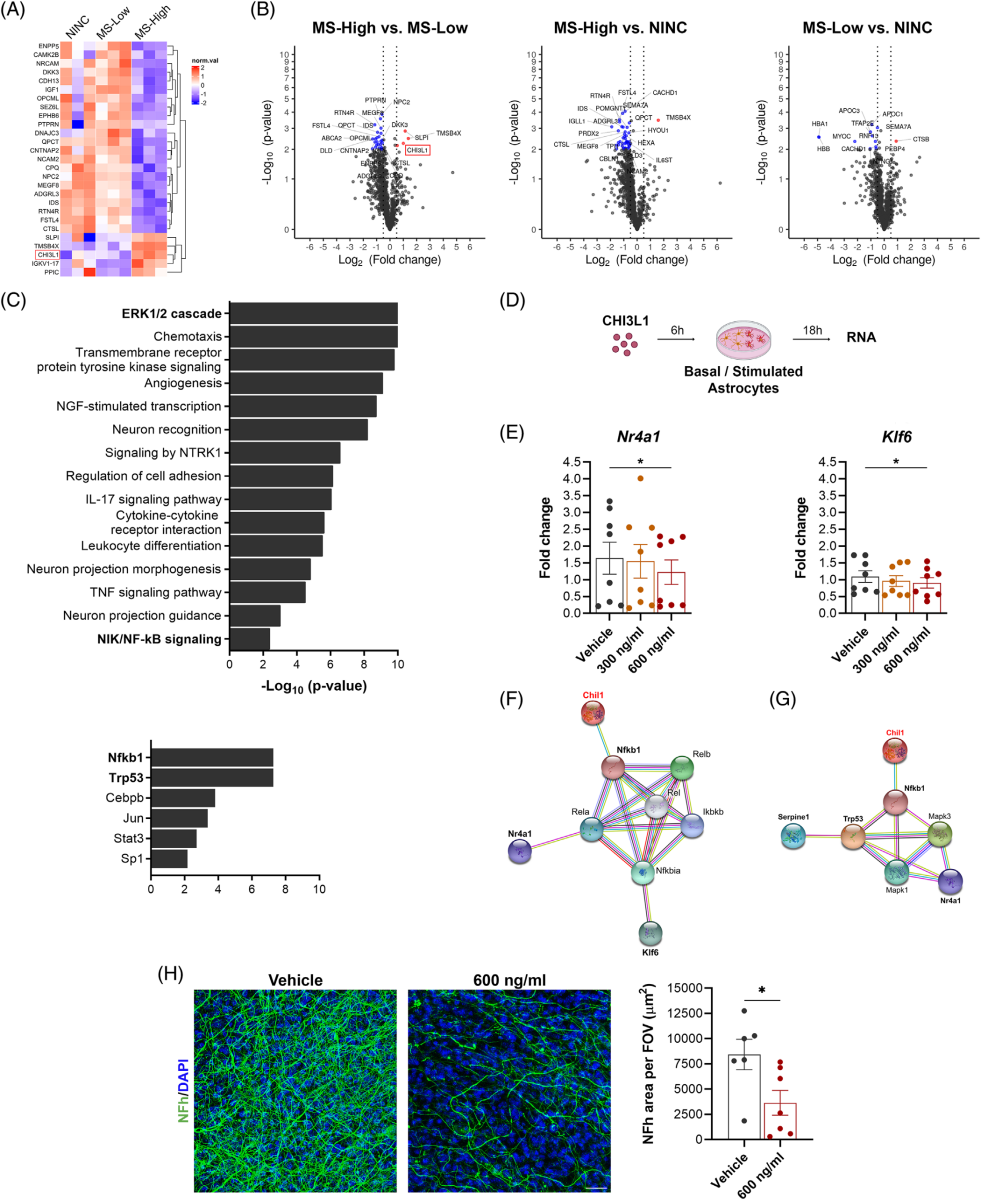
CHI3L1 is a potential mediator of the reactive astrocyte state by enhancing the pro‐inflammatory NF‐ĸB signalling pathway. (A, B) Liquid chromatography/mass spectrometry analysis of CSF from MS patients with inflammatory activity (MS‐High; *N* = 9), low inflammatory activity (MS‐Low; *N* = 9) and non‐inflammatory neurological controls (NINC; *N* = 9). (A) Heatmap and (B) volcano plots showing normalised Log_2_ protein expression satisfying *p*‐value < .01 and absolute *FC *> 0.5 identifying upregulation of CHI3L1 in MS‐High CSF (inset). (C) Plots showing bioinformatic annotation analysis integrating CSF, reactive astrocytes and astrocytic secretome data sets of the MS‐High condition. ERK1/2 cascade (*p = *4.7 × 10^–11^), NIK/NF‐ĸB signalling (*p = *.004) and *Nfkb1* (*FDR *= 5.3 × 10^–8^) exhibit substantial changes, pointing to enhanced NF‐ĸB signalling. (D) Flowchart illustrating primary purified astrocyte cultures stimulated with either PBS (Vehicle) or CHI3L1 at 300 and 600 ng/ml, using the same exposure conditions as previously in the CSF stimulation experiments. (E) qPCR assessment of CHI3L1 stimulation. The 600 ng/ml CHI3L1 concentration induces a reduced expression of *Nr4a1* (*p = *.03) and *Klf6* (*p = *.02). Individual values represent average *FC *= 2^–(average ∆∆Ct)^ in mRNAs in CHI3L1‐stimulated astrocytes relative to vehicle. Least Squares Means Estimates test and Tukey–Kramer multiple comparisons test; *n *= 8 independent biological samples per group. (F) Network diagram of differentially regulated contributors of enhanced NF‐ĸB signalling in MS‐High‐exposed reactive astrocytes predict CHI3L1 as an upstream regulator of astrocyte reactivity (*p = *3.5 × 10^–5^). (G) A node of interaction between *Mapk3* (ERK1), *Mapk1* (ERK2), *Trp53* and *Nr4a1* in the NF‐ĸB transcription module may potentially be regulated by CHI3L1 and would also affect *Serpine1* expression (*p = *.0002). (H) Illustrative confocal images of P7 murine organotypic brain slices treated at 7 DIV with either PBS (Vehicle) or CHI3L1 (600 ng/ml) for 48 h. Neurons were immunostained with neurofilament heavy chain (NFh, green) and DAPI (blue). Graphs represent individual data of averaged NFh area (μm^2^) per stack in each field of view (FOV). Least Squares Means Estimates test; *n *= 7 mice (Vehicle, *n *= 6). Data in E and H are shown as mean (SEM). *p < .05. Scale bar: 30 μm (H). DIV: days in vitro

Remarkably, we identified the prognostic biomarker chitinase 3‐like 1 (CHI3L1)^10^ upregulated in the MS‐High CSF (Figure 4B) and represented in the aforementioned pathways (Table S3). To investigate whether CHI3L1 could be a mediator of the MS‐High reactive astrocyte state, we stimulated astrocytes with CHI3L1 at concentrations above the cut‐off value that demonstrated prognostic implications in MS patients^10^ (Figure 4D). CHI3L1 stimulation (600 ng/ml) downregulated *Nr4a1* and *Klf6* expression, both involved in the inhibition of NF‐ĸB signalling^5^, ^6^ (Figure 4E and Figure S3). Protein interactome computation revealed an interaction between the NF‐ĸB transcription module (*Nr4a1* and *Klf6*) potentially controlled by CHI3L1 (Figure 4F). Noteworthy, we also found interactions between *Mapk3* (ERK1), *Mapk1* (ERK2) and *Nr4a1* in the NF‐ĸB transcription module, which might be regulated by CHI3L1 and SerpinE1 (Figure 4G). Finally, to address whether CHI3L1 could be a potential driver of astrocyte‐mediated neuronal damage we used P7 murine myelinating organotypic brain slice cultures that generate compact myelin ex vivo and mimic in vivo microenvironment. After 48 h, CHI3L1 (600 ng/ml) induced axonal damage reducing total neurofilament area (Figure 4H).

Our findings provide evidence that the degree of inflammatory activity in MS patients at disease onset has the potential to induce a specific reactive state in astrocytes that trigger neuronal damage (Figure S4). This reactive state, mainly associated with the NF‐ĸB signalling, could be exploited as a prognostic biomarker that reflects a potential detrimental effect of MS astrocytes on neuronal plasticity.

## CONFLICT OF INTEREST

The authors report no competing interests. G.G.D.B. is now an employee of Evotec.

## FUNDING INFORMATION

The study was funded by the ‘Red Española de Esclerosis Múltiple (REEM)’ sponsored by the ‘Fondo de Investigación Sanitaria’ (FIS; project reference: PI15/01111), Ministry of Science and Innovation, Spain; the ‘Ajuts per donar Suport als Grups de Recerca de Catalunya’, sponsored by the ‘Agència de Gestió d'Ajuts Universitaris i de Recerca’ (AGAUR), Generalitat de Catalunya, Spain; and Wellcome Trust (110138/Z/15/Z, to D.C.F.), United Kingdom.

## Supporting information

Table S1. Demographic data of the MS patients and controls included in the study
Figure S1. Astrocytes exposed to CSF from MS patients with high inflammatory activity exhibit an altered pro‐inflammatory secretome
Table S2. GO and KEGG functional analysis for the secretome content of astrocytes exposed to the MS‐High compared to the MS‐Low condition
Figure S2. Astrocytes exposed to MS‐High‐derived CSF exhibit a pro‐inflammatory signature mainly associated with NF‐kB signalling pathway
Table S3. Bioinformatic analysis integrating the MS‐High‐exposed astrocyte‐specific fingerprint compared to the MS‐Low exposure
Figure S3. Expression levels of genes associated with the specific astrocyte‐derived gene expression signature following CHI3L1 stimulation
Figure S4. Schematic flowchart summarising the main results of the study
