# Supplementary material for: Inflammation in multiple sclerosis induces a specific reactive astrocyte state driving non‐cell‐autonomous neuronal damage
Source: Clin Transl Med. 2022 May 11;12(5):e837. doi: 10.1002/ctm2.837 (PMC9091996; doi:10.1002/ctm2.837)
Supplement: Supplementary file 1 — Table S1. Demographic data of the MS patients and controls included in the study Figure S1. Astrocytes exposed to CSF from MS patients with high inflammatory activity exhibit an altered pro‐inflammatory secretome Table S2. GO and KEGG functional analysis for the secretome content of astrocytes exposed to the MS‐High compared to the MS‐Low condition Figure S2. Astrocytes exposed to MS‐High‐derived CSF exhibit a pro‐inflammatory signature mainly associated with NF‐kB signalling pathway Table S3. Bioinformatic analysis integrating the MS‐High‐exposed astrocyte‐specific fingerprint compared to the MS‐Low exposure Figure S3. Expression levels of genes associated with the specific astrocyte‐derived gene expression signature following CHI3L1 stimulation Figure S4. Schematic flowchart summarising the main results of the study [file CTM2-12-e837-s001.docx]

# Supporting information

| Table S1. Demographic data of the MS patients and controls included in the study. | | | | |
| --- | --- | --- | --- | --- |
|  | **Age (years)** | **Female/male (% females)** | **Time LP-MRI (years)** | **Corticosteroid treatment (%)** |
| Optimization cohort | | |  |  |
| MS-High | **25.6 (8.0)** | **4/0 (100.0)** | **0.1 (0.02-0.20)** | **0/0 (0.0)** |
| Pool-1 | 22.0 (3.3) | 2/0 (100.0) | 0.1 (0.00-0.22) | 0/0 (0.0) |
| Pool-2 | 29.2 (11.4) | 2/0 (100.0) | 0.1 (0.05-0.15) | 0/0 (0.0) |
|  |  |  |  |  |
| Final cohort | | |  |  |
| MS-High | **29.3 (6.2)** | **7/2 (77.8)** | **0.2 (0.05-0.41)** | **1/9 (11.1)** |
| Pool-1 | 24.7 (4.7) | 2/1 (66.7) | 0.1 (0.03-0.22) | 1/3 (33.3) |
| Pool-2 | 27.3 (4.5) | 2/1 (66.7) | 0.3 (0.06-0.42) | 0/3 (0.0) |
| Pool-3 | 36.0 (2.0) | 3/0 (100.0) | 0.4 (0.13-0.49) | 0/3 (0.0) |
| MS-Low | **35.4 (8.0)** | **6/3 (66.7)** | **0.2 (0.12-0.23)** | **1/9 (11.1)** |
| Pool-1 | 43.3 (8.5) | 2/1 (66.7) | 0.2 (0.11-0.20) | 1/3 (33.3) |
| Pool-2 | 34.0 (4.6) | 2/1 (66.7) | 0.1 (0.11-0.19) | 0/3 (0.0) |
| Pool-3 | 29.0 (1.7) | 2/1 (66.7) | 0.3 (0.16-0.28) | 0/3 (0.0) |
| NINC | **42.0 (11.7)** | **8/1 (88.9)** | **-** | **-** |
| Pool-1 | 30.0 (10.4) | 3/0 (100.0) | **-** | **-** |
| Pool-2 | 52.0 (6.9) | 3/0 (100.0) | **-** | **-** |
| Pool-3 | 44.0 (4.6) | 2/1 (66.7) | **-** | **-** |

Age is expressed as mean (standard deviation). Time between lumbar puncture (LP) and baseline brain MRI is expressed as median (interquartile range). Numbers in bold represent the mean values of all the pools included in each group of patients and controls. MS-High/Low: MS patients with high and low inflammatory activity, respectively. MS-High: high inflammatory multiple sclerosis; MS-Low: low inflammatory multiple sclerosis; NINC: non-inflammatory neurological disease controls.

**
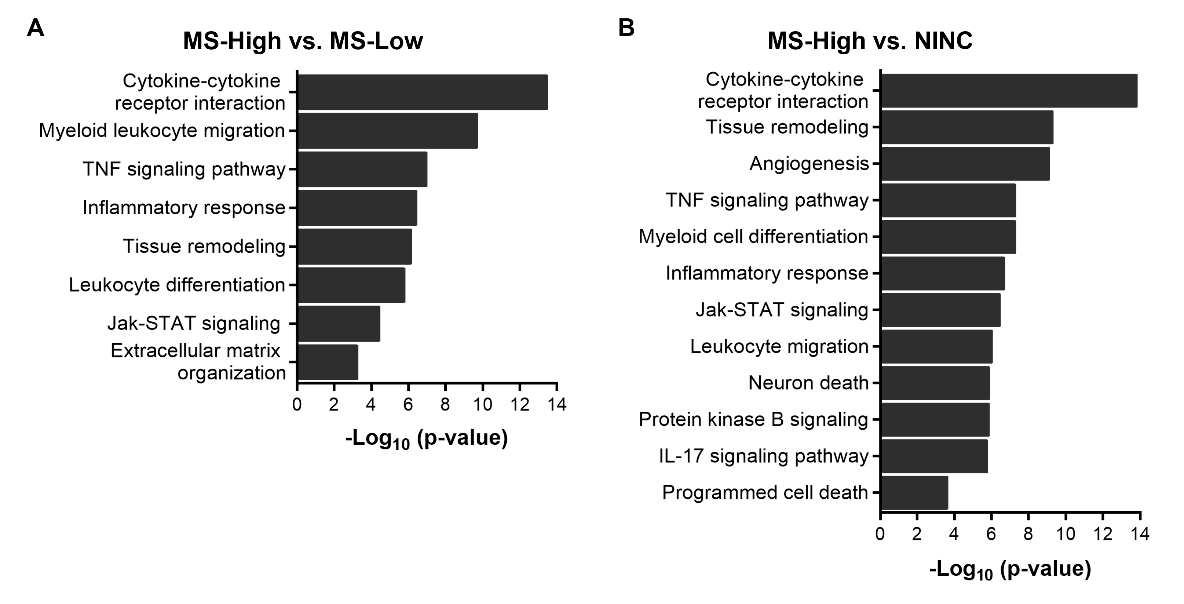
**

**Figure S1. Astrocytes exposed to CSF from MS patients with high inflammatory activity exhibit an altered pro-inflammatory secretome**. Plots showing GO and KEGG functional annotation analysis for the MS-High condition compared to the MS-Low (**A**) and non-inflammatory control (NINC) conditions (**B**).

| Table S2. GO and KEGG functional analysis for the secretome content of astrocytes exposed to the MS-High compared to the MS-Low condition. | | |
| --- | --- | --- |
| Pathways | **FDR** | **Genes** |
| Cytokine-cytokine receptor interaction | 3.1 x 10^-14^ | *Csf1, Cxcl1, Il11, Lep, Lif, Pdgfb, Ccl2, Ccl19,* ***Serpine1****, Igfbp5, Mmmp2* |
| Myeloid leukocyte migration | 1.9 x 10^-10^ | *Csf1, Cxcl1,* ***Serpine1****, Pdgfb, Ccl12, Ccl19, Lep, Igfbp5* |
| TNF signaling pathway | 9.9 x 10^-8^ | *Csf1, Cxcl1, Lif, Ccl12* |
| Inflammatory response | 3.5 x 10^-7^ | *Csf1, Cxcl1, Lep,* ***Serpine1****, Ccl12, Ccl19* |
| Tissue remodeling | 7.0 x 10^-7^ | *Igfbp5, Lep, Lif, Mmp2, Csf1, Pdgfb* |
| Leukocyte differentiation | 1.6 x 10^-6^ | *Csf1, Il11, Lep, Lif, Ccl19, Pdgfb* |
| Jak-STAT signaling | 3.5 x 10^-5^ | *Il11, Lep, Lif* |
| Extracellular matrix organization | 0.0005 | *Mmp2,* ***Serpine1****, Pdgfb* |

Functional annotation enrichment of astrocyte-secreted factors from astrocytes exposed to the MS-High condition compared to the MS-Low condition. SerpinE1 is highlighted in bold in those pathways in which is involved. Mouse gene ID symbols are shown. Csf1: M-CSF; Lep: leptin.

**
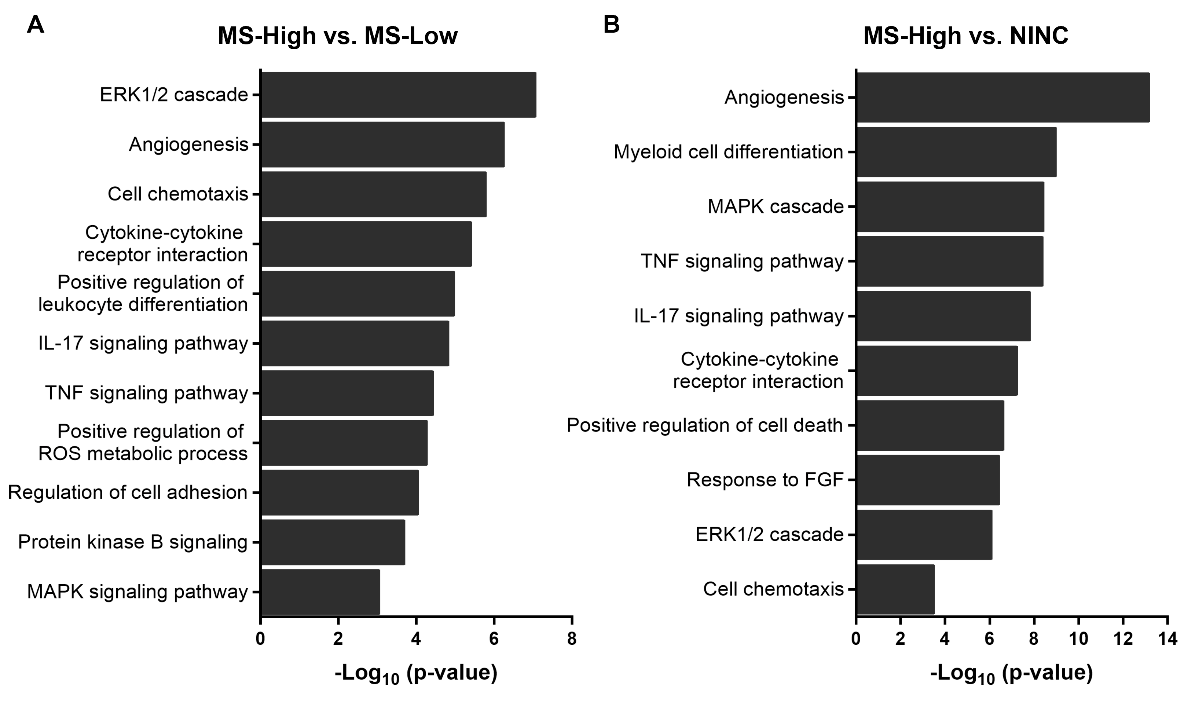
**

**Figure S2. Astrocytes exposed to MS-High-derived CSF exhibit a pro-inflammatory signature mainly associated with NF-kB signalling pathway.** Plots showing functional annotation enrichment analysis integrating transcriptomic, proteomic and secretomic data of MS-High compared to MS-Low (**A**) and NINC (**B**)- exposed reactive astrocytes.

| Table S3. Bioinformatic analysis integrating the MS-High-exposed astrocyte-specific fingerprint compared to the MS-Low exposure. | | |
| --- | --- | --- |
| Pathways | **FDR** | **Genes** |
| ERK1/2 cascade | 4.7 x 10^-11^ | *ATF3, CASR,* ***CHI3L1****, DUSP1, DUSP6, EPHB2, HMGB1, IGF1, KDR, LIF, PDGFB, CCL12, CCL19, SPRY1, SPRY2, SPRY4, DUSP5, IL11, LEP, SORL1, GDF15, DDR1, CSF1, EGR1, EPHB6, ARRDC3, IRGM, IGFBP5, AKR1C3* |
| Chemotaxis | 7.9 x 10^-11^ | *CASR, CDH13, CSF1, DUSP1, MEGF8, EGR2, EGR3, EPHB2, EPHB6, CXCL1, HMGB1, NR4A1, KDR, NRCAM,* ***SERPINE1****, PDGFB, CCL12, CCL19, TMSB4X, EMB, SEMA3D* |
| Transmembrane receptor protein tyrosine kinase signaling pathway | 1.6 x 10^-10^ | *DDR1, CDH13, CSF1, EPHB2, EPHB6, IGF1, IGFBP5, KDR, LEP, MMP2, PDGFB, PRL, SORL1, OGT, GDF15, SPRY1, SPRY2, TXNIP, FSTL4, DDIT4, CSRNP1, SPRY4, LPAR6, RTN4R, PLK2* |
| Angiogenesis | 7.8 x 10^-10^ | *CDH13,* ***CHI3L1****, EGR3, EPHB2, HMGB1, NR4A1, KDR, LEP, MMP2, NRCAM,* ***SERPINE1****, PRL, PTGS2, CCL12, SP1, ADAMTS1, SPRY2, KLF2, PLK2* |
| NGF-stimulated transcription | 1.8 x 10^-9^ | *EGR1, EGR2, EGR3, FOS, FOSB, SGK1, ARC* |
| Neuron recognition | 6.3 x 10^-9^ | *MEGF8, EPHB2, NCAM2, NRCAM, OPCML, CNTNAP2, EMB, CCL19, CAMK2B, CSF1, CSNK1D, EGR2, IGF1, LIF* |
| Signaling by NTRK1 (TRKA) | 2.7 x 10^-7^ | *DUSP6, EGR1, EGR2, EGR3, FOS, FOSB, SGK1, ARC* |
| Regulation of cell adhesion | 7.5 x 10^-7^ | *DDR1, CDH13, CSF1, DUSP1, EGR3, HMGB1, IGF1, KDR, LEP, LIF,* ***SERPINE1****, PDGFB, CCL12, CCL19, SASH3, NFKBIZ, SPRY4, FOS, KLF6, CTSL, EGR1, IL11, ZFP36,* ***CHI3L1****, TMSB4X, TBK1, PSMD4, PTGS2, SORL1, KLF2* |
| IL-17 signaling pathway | 8.9 x 10^-7^ | *FOS, FOSB, CXCL1, LCN2, PTGS2, CCL12, TBK1* |
| Cytokine-cytokine receptor interaction | 2.4 x 10^-6^ | *CSF1, CXCL1, IL11, KDR, LEP, LIF, PDGFB, PRL, CCL12, CCL19, GDF15* |
| Leukocyte differentiation | 2.9 x 10^-6^ | *CSF1, EGR3, FOS, HMGB1, LIF, CCL19, SASH3, NFKBIZ* |
| Neuron projection morphogenesis | 1.5 x 10^-5^ | *DDR1, CAMK2B, MEGF8, EGR2, EPHB2, EPHB6, NRCAM, SGK1, FSTL4, ARC, CNTNAP2, RTN4R, EMB, SEMA3D* |
| TNF signaling pathway | 3.2 x 10^-5^ | *CSF1, FOS, CXCL1, LIF, PTGS2, CCL12* |
| Neuron projection guidance | 0.0009 | *MEGF8, EGR2, EPHB2, EPHB6, NRCAM, EMB, SEMA3D* |
| NIK/NF-kB signaling | 0.004 | ***CHI3L1****, HMGB1, PSMD4, CCL19, TMSB4X* |

Bioinformatic functional annotation analysis integrating CSF, reactive astrocytes and astrocytic secretome data sets of the MS-high condition. SERPINE1 and CHI3L1 are highlighted in bold. Human gene ID symbols are shown. *CSF1*: M-CSF. *LEP*: leptin.

**Figure S3. Expression levels of genes associated with the specific astrocyte-derived gene expression signature following CHI3L1 stimulation.** Primary purified astrocyte cultures were stimulated with either PBS (Vehicle) or CHI3L1 at 300 and 600 ng/ml as previously described. mRNA expression levels were measured by qPCR. Individual values represent average *FC* = 2^- (average ∆∆Ct)^ in mRNAs in CHI3L1-stimulated astrocytes relative to the vehicle condition. Least Squares Means Estimates test and Tukey-Kramer multiple comparisons test; *n*=8 independent biological samples per group. Data are shown as mean (SEM). **P*<0.05.


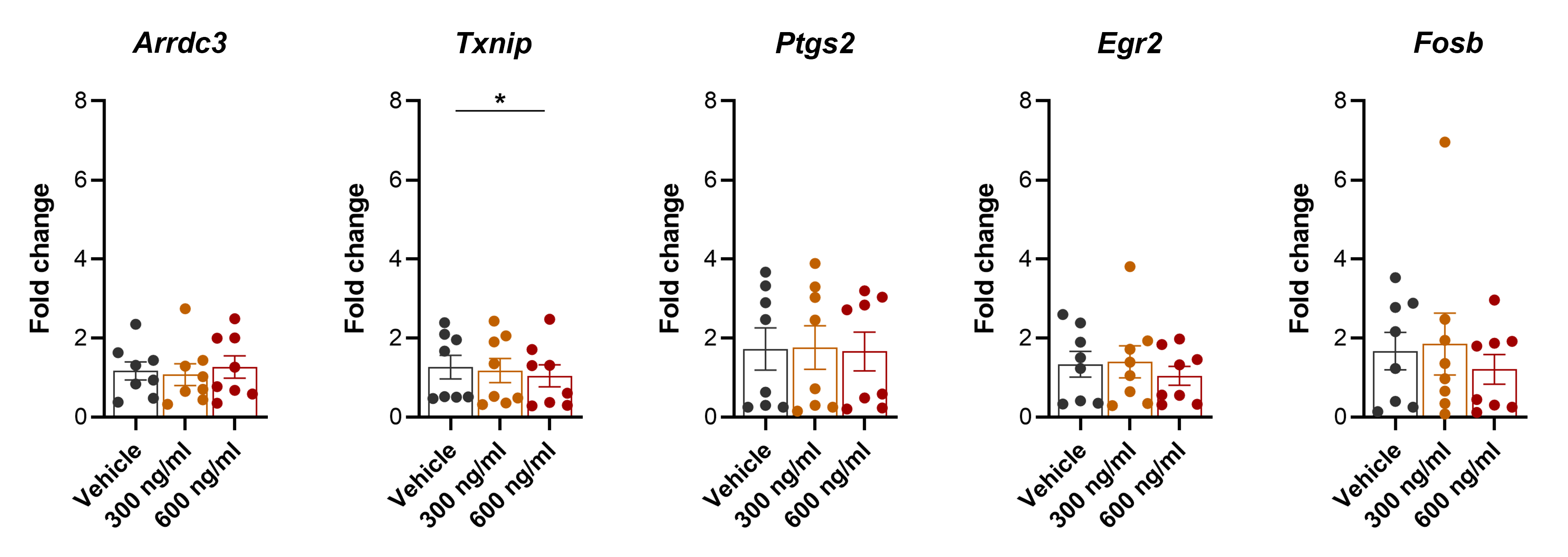


**
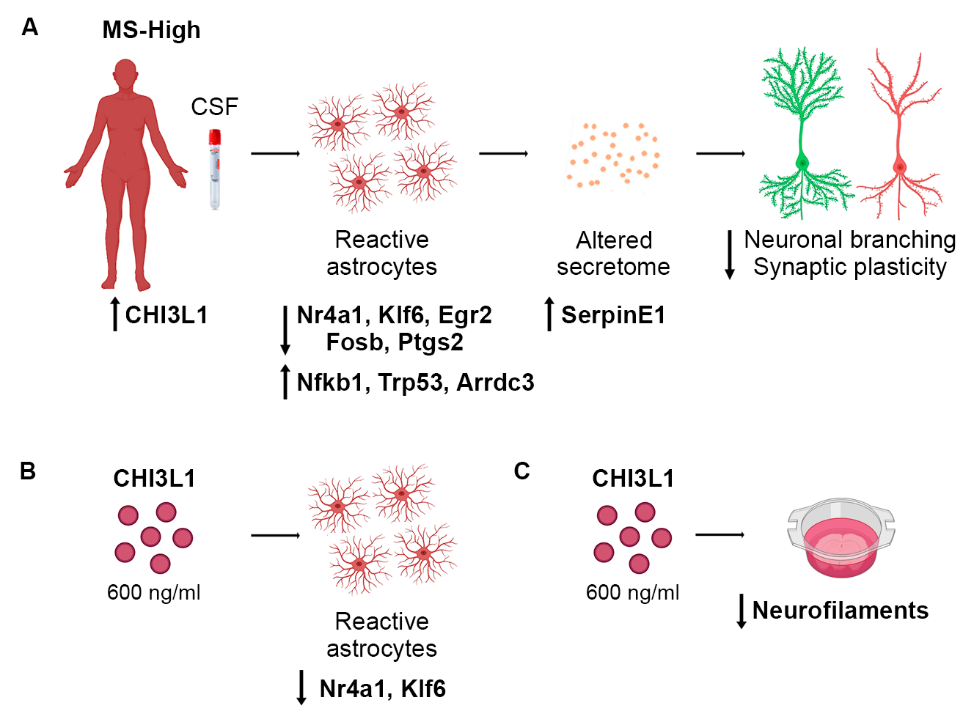
**

**Figure S4. Schematic flowchart summarizing the main results of the study.** CSF from MS patients with high inflammatory activity (MS-High) induces a specific reactive astrocyte state *in vitro* characterized by enhanced NF-ĸB activation, which confers a maladaptive response accompanied by an altered secretome that leads to neuronal morphological alterations and synaptic plasticity impairment. SerpinE1 up-regulated in astrocytic secretomes is a potential downstream mediator of non-cell-autonomous neuronal damage. In addition, chitinase 3-like 1 (CHI3L1), increased in CSF from MS-High patients, is a potential upstream modulator of astrocyte reactivity and a potential driver of astrocyte-mediated neuronal damage.
